# Key factors influencing children’s cooperation during electromyography: a correlation analysis of Age, gender, and pain management

**DOI:** 10.3389/fped.2026.1819172

**Published:** 2026-04-13

**Authors:** Jing Xie, Feng Han, Yujia Chen, Xiaochen Zhu, Zhili Rong, Jiao Lin, Hongzheng Chen, Yichao Xu, Yunqing Zhou

**Affiliations:** 1 Department of Rehabilitation, Shanghai Children’s Medical Center, Shanghai Jiao Tong University School of Medicine, Shanghai, China; 2 Department of Neurology, Shanghai Children’s Medical Center, Shanghai Jiao Tong University School of Medicine, Shanghai, China; 3 Hainan Branch, Shanghai Children’s Medical Center, Shanghai Jiao Tong University School of Medicine, Shanghai, China

**Keywords:** age, gender, needle electrode, pain, pediatric electromyography, procedural cooperation

## Abstract

**Objective:**

To investigate the influence of age, gender, and pain level on cooperation during electromyography (EMG) examinations in children, and to provide insights that may inform the optimization of pain management and operational strategies for pediatric EMG.

**Methods:**

A total of 44 children who underwent EMG examination at Shanghai Children's Medical Center, Shanghai Jiao Tong University School of Medicine from November 2024 to November 2025 were enrolled. Data on age, gender, Visual Analogue Scale (VAS) pain score, and physician-assessed cooperation level were collected. Spearman correlation analysis and multiple linear regression analysis were employed to evaluate the relationship between these factors and the cooperation level.

**Results:**

The mean age of the children was 10.36 ± 3.13 years, with 22 males and 22 females. The mean VAS pain score was 5.30 ± 2.61, and the mean cooperation score was 3.93 ± 1.04. Correlation analysis revealed a significant positive correlation between age and cooperation level (r_s_ = 0.593, *p* < 0.001), and a significant negative correlation between VAS score and cooperation level (r_s_ = −0.693, *p* < 0.001).No significant correlation was found between gender and cooperation level (r_s_ = −0.232, *p* = 0.113). Multiple regression analysis confirmed that both age and VAS score were independent factors affecting cooperation level (R^2^ = 0.634, *p* < 0.001).

**Conclusion:**

Age and pain level are key determinants of cooperation during EMG examinations in children. Our findings suggest that pain management strategies could benefit from incorporating individualized analgesia protocols, psychological interventions, and future optimization of electrode technology to enhance examination compliance and diagnostic quality in pediatric patients.

## Introduction

Electromyography, an essential electrophysiological technique for diagnosing neuromuscular diseases, plays an irreplaceable role in pediatric clinical practice (1). However, its invasive nature often induces significant pain, which is particularly pronounced in children. Due to their immature nervous systems and limited psychological and cognitive capacities, pediatric patients generally exhibit lower pain tolerance, frequently manifesting as anxiety, fear, and poor cooperation. These reactions can significantly impede the examination process and compromise the reliability of results (1–3).

Studies indicate that pain perception during EMG and nerve conduction studies in children is variable and closely associated with factors such as age, number of muscles examined, and analgesic protocols used (1). Furthermore, psychological factors such as anxiety and catastrophic thinking significantly modulate the pain experience (2, 4, 5). Therefore, systematically evaluating the key factors affecting children's cooperation during EMG and formulating individualized pain management strategies based on these findings holds significant clinical importance.

The concentric needle electrode, widely used in EMG examinations, has specifications and material properties that directly influence puncture pain and signal quality (6, 7). Most existing electrodes are designed for adults, lacking specialized models tailored to children's physiological characteristics, often resulting in inadequate pain control or unstable signal acquisition in clinical applications (8, 9). Additionally, research on the relationship between age, gender, and pain perception remains limited, particularly regarding their mechanistic roles in children's cooperation during EMG.

This study analyzed clinical data from 44 children undergoing EMG to explore the impact of age, gender, and pain scores on examination cooperation. Combined with a review of recent literature on pain mechanisms, electrode design, and non-pharmacological interventions, it aims to provide a theoretical basis and practical guidance for optimizing pediatric EMG examinations, promoting their development toward greater safety, comfort, and efficiency.

## Materials and methods

### Study population

This cross-sectional study enrolled children who underwent EMG examination in the Department of Neurology, Shanghai Children's Medical Center, Shanghai Jiao Tong University School of Medicine, between November 2024 to November 2025. Inclusion criteria were: age 5 to 18 years; ability to understand and follow basic instructions during the examination. Exclusion criteria included: severe cognitive or communication impairment preventing understanding of the research or completion of pain assessment; incomplete clinical data. A total of 44 children were included, and informed consent was obtained from all guardians. This study was approved by the Ethics Committee of Shanghai Children's Medical Center (Approval No.SCMCIRB-K2025292-1).

The clinical indications for EMG in this cohort primarily included suspected neuromuscular disorders (e.g., myopathy, neuropathy), focal nerve injuries, and radiculopathies.

### Examination equipment and electrodes

All electrophysiological examinations were performed using the Alpine Keypoint9 EMG/Nerve Conduction Velocity Measuring Instrument (Model: 033407). The EMG examination utilized Dantec concentric needle electrodes (Model: 9013S0032), with the following specifications: needle length 37 mm, diameter 0.46 mm (26G), recording area 0.07 mm^2^. This electrode model offers excellent conductivity and mechanical strength and is commonly used in clinical practice (6, 7).

### Examination procedure and pain/ cooperation assessment

All EMG examinations were performed by two designated physicians with extensive experience in pediatric electrophysiology to minimize inter-operator variability.

Immediately following the examination, the research nurse instructed the child to rate the overall pain level using the Visual Analogue Scale (VAS), ranging from 0 (“no pain”) to 10 (“most intense pain imaginable”). For younger children or those with difficulty understanding the VAS, facial expression cards were used as supplementary aids to ensure valid scoring (10). The child's cooperation level was independently assessed by the two examining physicians using a 5-point scale. This scale was developed based on clinical consensus for the purpose of this study and has not undergone formal external validation. The final cooperation score was the average of the two physicians' ratings. Inter-rater reliability was assessed using the Intraclass Correlation Coefficient (ICC), which yielded a value of 0.89 (95% CI: 0.82–0.94), indicating excellent agreement between the raters. 1: Completely uncooperative, examination not feasible; 2: Mostly uncooperative, requires frequent interruption/reassurance, low completion; 3: Moderately completion, requires some reassurance, completes basic items; 4: Mostly cooperative, slight resistance during some maneuvers; 5: Fully cooperative, quiet and cooperative throughout. The final cooperation score was the average of the two physicians' ratings.

### Statistical analysis

Data were analyzed using SPSS 29.0 (IBM Corp., Armonk, NY). Normally distributed continuous data are presented as mean ± standard deviation, non-normally distributed data as median (interquartile range), and categorical data as number (percentage). Spearman rank correlation analysis was used to assess correlations between age, gender, VAS score, and cooperation level. Multiple linear regression analysis (forward selection) was performed with cooperation level as the dependent variable and age, gender, and VAS score as independent variables to identify independent predictors. Multicollinearity was assessed using the variance inflation factor (VIF), with VIF < 10 considered acceptable. Statistical significance was set at *α* = 0.05 (two-tailed); *p* < 0.05 was considered significant.

## Results

### Baseline characteristics and descriptive statistics

All 44 enrolled children were included in the analysis. Baseline characteristics, pain scores, and cooperation scores are summarized in Table 1. The children's ages ranged from 5 to 18 years, with a mean of 10.36 ± 3.13 years. Gender distribution was equal (22 males, 22 females). The mean VAS pain score was 5.30 ± 2.61 [median [IQR]: 5 [4.00]], indicating moderate pain. The mean cooperation score was 3.93 ± 1.04 [median [IQR]: 4 [2.00]], suggesting that most children were generally cooperative and completed the examination.

**Table 1:** Demographic and clinical characteristics of the study population (*N* = 44).

Characteristic	Value
Age (years), mean ± SD	10.36 ± 3.13
Gender, *n* (%)	
Male	22（50.0%）
Female	22（50.0%）
VAS Score, mean ± SD	5.30 ± 2.61
VAS Score, median (IQR)	5（4.00）
Cooperation Score, mean ± SD	3.93 ± 1.04
Cooperation Score, median (IQR)	4（2.00）

VAS, visual analogue scale; SD, standard deviation; IQR, interquartile range.

### Correlation analysis between factors and cooperation level

**Figure 1:**
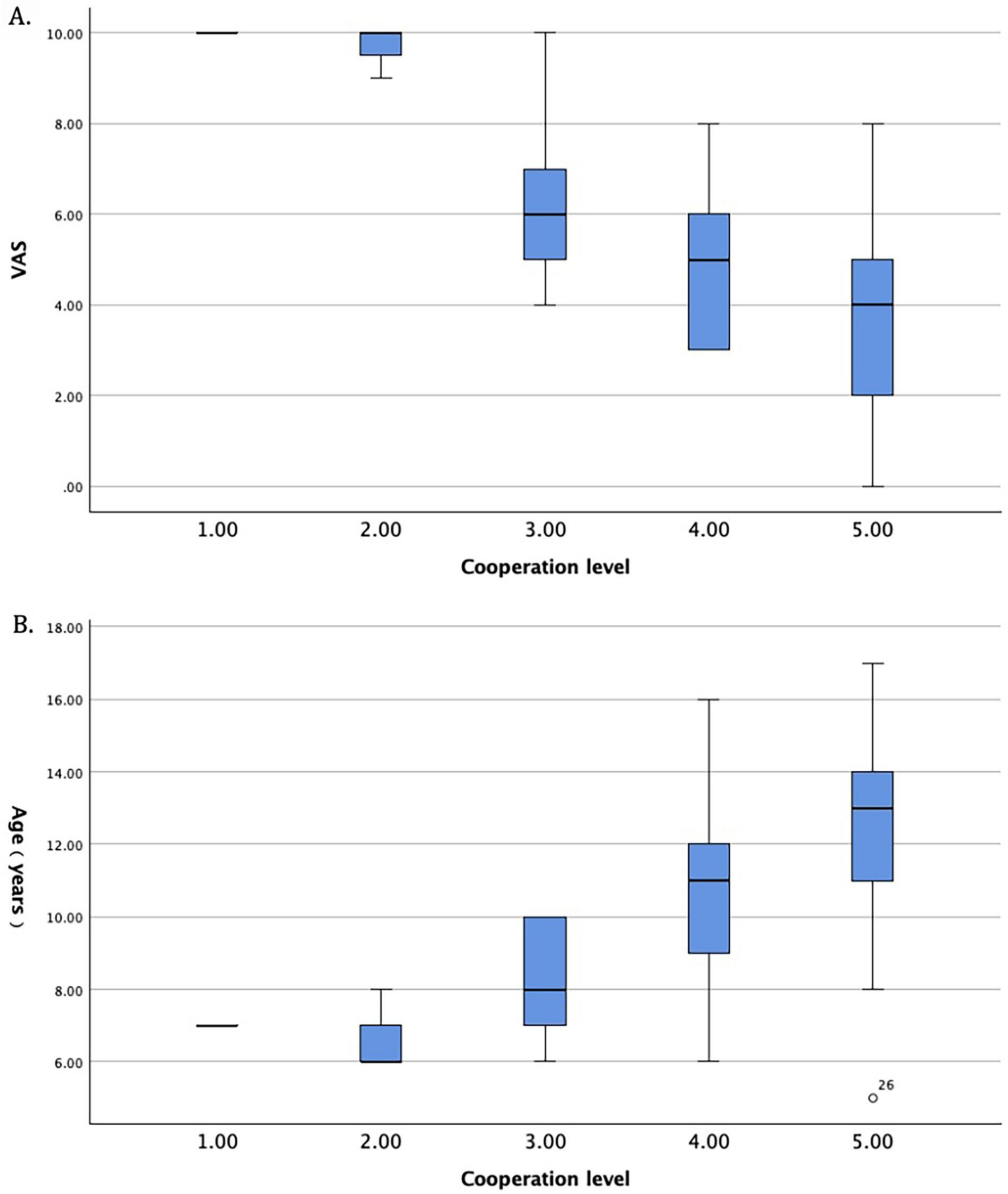
Relationship between cooperation level during EMG examination and pain score or age in children. **(A)** Box plot comparing Visual Analogue Scale scores across different cooperation levels. A significant negative correlation was observed between cooperation level and VAS pain score (r_s_ = −0.693, *p* < 0.001). The box represents the interquartile range, the line inside the box indicates the median, and the whiskers show the minimum and maximum values. **(B)** Box plot showing the distribution of age across different cooperation levels. A significant positive correlation was observed between cooperation level and patient age (r_s_ = 0.593, *p* < 0.001).

**Figure 2:**
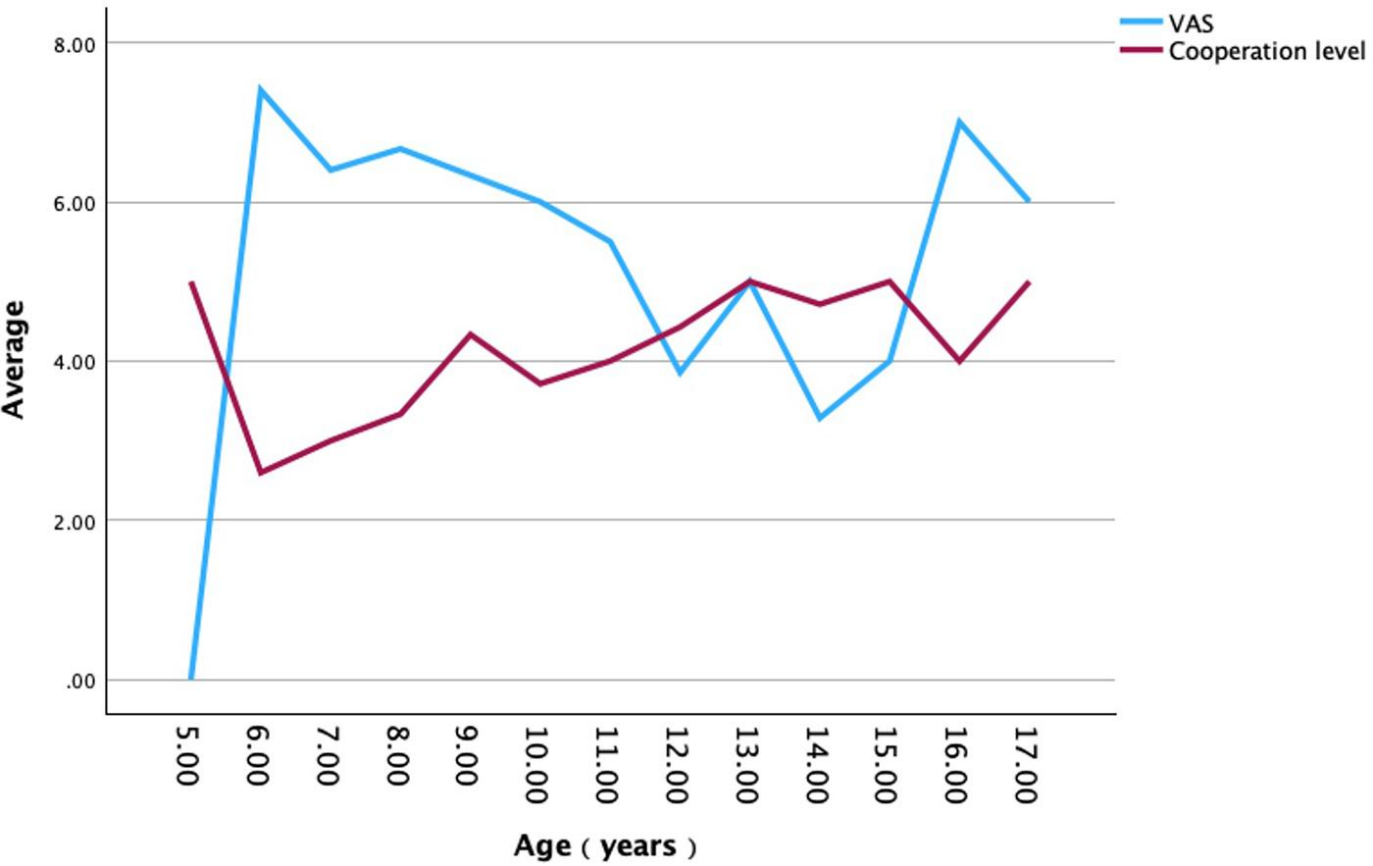
Relationship between age and pain, cooperation level. This line chart illustrates the trend of the relationship between patient age and Visual Analogue Scale pain scores, as well as examination cooperation level. X-axis: Age (years). Y-axes: Left axis shows the VAS pain score (sky blue line), ranging from 0 to 10. Right axis shows the examination cooperation level score (burgundy line), ranging from 1 to 5.

Spearman correlation analysis (Table 2) demonstrated a significant positive correlation between age and cooperation level (Figure 1) (r_s_ = 0.593, *p* < 0.001), indicating higher cooperation in older children. A significant negative correlation was observed between VAS pain score and cooperation level (Figure 2) (r_s_ = −0.693, *p* < 0.001), suggesting that greater pain was associated with poorer cooperation. No significant correlation was found between gender and cooperation level (r_s_ = −0.232, *p* = 0.113).

**Table 2:** Correlation analysis of Age, gender, VAS score with cooperation level.

Factor	r_s_ with cooperation level	*p*-value
Gender	−0.242	0.113
Age	0.593	0.000**
VAS	−0.693	0.000**

VAS, visual analogue scale; CI, confidence interval; SD, standard deviation.

** *p* < 0.01.

### Multiple linear regression analysis of factors affecting cooperation level

Multiple linear regression analysis (forward method) was conducted with cooperation level as the dependent variable and age, gender, and VAS score as independent variables. The gender variable was excluded from the final model. The regression model was statistically significant (F = 35.461, *p* < 0.001), with an adjusted R^2^ of 0.634, indicating that age and VAS score collectively explained 63.4% of the variance in cooperation level.

As shown in Table 3, age was an independent positive predictor of cooperation level (*β* = 0.138, *p* < 0.001), while VAS pain score was an independent negative predictor (*β* = −0.224, *p* < 0.001). The VIF values for both predictors were 1.113, indicating no significant multicollinearity.

**Table 3:** Multiple linear regression analysis of factors affecting children's cooperation level.

Factor	t-value	*p*-value	VIF	B (95% CI)
Age	4.153	<0.001	1.113	0.138（0.071, 0.205）
VAS	−5.623	<0.001	1.113	−0.224（−0.304, −0.144）
(Constant)	7.872	<0.001	/	3.686 (2.740, 4.632)

VAS, visual analogue scale; CI, confidence interval; VIF, variance inflation factor; SD, standard deviation.

## Discussion

This study systematically explored the relationship between age, gender, pain cooperation level during EMG examinations in 44 children. The primary findings confirm that age and pain perception are two independent key factors influencing children's cooperation, whereas gender did not demonstrate a significant effect in this cohort. These results provide important empirical evidence for developing targeted optimization strategies in clinical practice.

The VAS pain score showed a significant negative correlation with cooperation level (rs = −0.693, *p* < 0.001), and emerged as the strongest negative predictor in the regression model (*β* = −0.224, *p* < 0.001). These results directly confirm clinical observations: pain during pediatric EMG leads to reduced cooperation, thereby potentially compromising examination accuracy and diagnostic value. EMG-related pain primarily arises from mechanical stimulation of the skin and muscle tissue by needle insertion, tissue damage, and secondary reactions from muscle contraction (1, 11). Children's immature nervous systems contribute to heightened pain sensitivity, and their pain experience is often compounded by negative emotions such as anxiety and fear, creating a vicious cycle (2, 3, 5). Therefore, effective pain management is paramount for improving cooperation.

The analgesic approach in this study primarily involved psychological reassurance during standard procedures, without systematic pharmacological intervention. However, literature suggests that individualized pharmacological analgesia, such as combined oral and topical agents tailored to the child's age and examination complexity, can significantly reduce pain scores (1). Furthermore, non-pharmacological interventions, including cognitive-behavioral therapy, distraction techniques (e.g., virtual reality), and optimizing the examination environment with parental presence, have proven effective in alleviating children's pain and anxiety (12–14). Future clinical practice should therefore implement evidence-based, multimodal pain management strategies integrating both pharmacological and non-pharmacological interventions to fundamentally enhance the examination experience and cooperation.

Age was significantly positively correlated with cooperation level (rs = 0.593, *p* < 0.001), and was an independent positive predictor (*β* = 0.138, *p* < 0.001). This aligns with established patterns of neuropsychological development. Older children typically possess more mature pain modulation mechanisms and greater cognitive capacity. They can better understand the examination's necessity, follow instructions for muscle relaxation and contraction, and employ more mature coping strategies for pain and stress (1, 15). Conversely, younger children, with less developed higher cognitive centers such as the prefrontal cortex, have a limited ability to regulate fear and pain, making them more prone to intense emotional and behavioral reactions and poorer cooperation (3, 10).

This finding has critical clinical implications. For younger children, thorough pre-examination preparation, frequent reassurance and encouragement during the procedure, and more proactive use of analgesia or sedation when necessary are particularly important (1, 16). It also suggests that suitability for EMG in children should be assessed not solely based on chronological age but also considering psychological maturity and communication ability.

This study utilized the Dantec 9013S0032 concentric needle electrode (diameter 0.46 mm). Although commonly used, the pain it induced significantly affected cooperation, raising a core question: how to balance comfort and diagnostic efficacy through technical optimization, particularly electrode innovation? The research team attempted using the thinner Dantec 9013S0012 electrode (diameter 0.30 mm) to reduce pain, but results were suboptimal. This model's excessively small recording area (0.02mm^2^) compromised signal quality. In our clinical practice and during the preparatory phase of this study, we observed that standard adult-sized concentric needle electrodes often caused significant discomfort. Furthermore, in cases where children exhibited strong involuntary muscle contractions due to pain, we noted instances of electrode deformation and damage. Although these specific deformation events were not quantitatively recorded as a primary outcome in this dataset, they represent a significant practical challenge. This observation underscores the urgent need for developing new specialized pediatric needle electrodes that carefully balance comfort, signal quality, and durability.

The ideal pediatric EMG electrode should be optimized in material, structure, and specifications. Material-wise, selection should favor high biocompatibility, appropriate stiffness, and excellent surface smoothness (e.g., nickel-titanium alloy or nano-coated stainless steel such as nano-silver) to reduce tissue damage, allergic risk, and consequently puncture pain (6, 7, 17, 18). Structurally, minimally invasive and intelligent design concepts should be adopted, such as optimizing needle tip geometry for easier penetration, or developing smart electrodes with integrated sensors for real-time feedback on signal quality and stimulation intensity (19–21). Regarding specifications, guidelines for model selection based on children's muscle volume and developmental stage are needed, prioritizing finer diameters and appropriate lengths while ensuring sufficient signal-to-noise ratio (8, 9). Future efforts should focus on standardizing specialized concentric needle electrodes for children, necessitating multidisciplinary collaboration among clinicians, materials scientists, and engineers (1).

Several limitations of this study must be acknowledged. First, the cooperation score was measured on an ordinal five-point scale but analyzed using linear regression, treating it as a continuous variable. While this approach is frequently employed in exploratory clinical research, we recognize that ordinal regression models might offer greater statistical rigor. Second, the cooperation assessment scale used was developed internally and lacks prior validation, although our high inter-rater reliability (ICC=0.89) supports its consistency within this context. Third, the relatively small sample size (*n* = 44) from a single center limits the statistical power and generalizability of our findings. Future multi-center studies with larger cohorts are needed to validate these results.

## Conclusion

This study confirms that a child's age and the pain level during EMG examination are key determinants of cooperation. To enhance the clinical value of pediatric EMG, it is essential to adopt evidence-based, multidimensional, and individualized strategies. This includes not only refining pharmacological and non-pharmacological pain management protocols but, crucially, also driving innovation in electrode technology to develop specialized concentric needle electrodes truly suited to children's physiological and psychological characteristics, ultimately achieving the dual goals of diagnostic accuracy and patient comfort.

## Data Availability

The raw data supporting the conclusions of this article will be made available by the authors, without undue reservation.
